# Assessment of the reproductive performances of native sows in Southern Benin

**DOI:** 10.1093/tas/txab089

**Published:** 2021-05-13

**Authors:** David Djimènou, Camus M Adoligbé, André B Aboh, Aubin G Amagnidé, Richard Osei-Amponsah, Aimé K Edénakpo, Pamphile C Tobada, Delphin O Koudandé

**Affiliations:** 1 National Agricultural Research Institute of Benin (INRAB), Cotonou, Bénin; 2 University of Abomey-Calavi (UAC), Abomey-Calavi, Benin; 3 National Agricultural University of Benin (UNA), Cotonou, Benin; 4 University of Ghana, Legon, Accra, Ghana

**Keywords:** conservation, farrowing interval, litter size, selection

## Abstract

Native sows contribute to a large extent to food security and poverty alleviation in Benin. However, their reproductive performance particularly under extensive systems is poorly characterized. The objective of this study was to fill this knowledge gap by selecting 284 multiparous sows based on hair color and some reproductive parameters. The results showed that native sows of Southern Benin can be clustered into three groups with group 3 showing the best reproductive performances including highest average litter size (LS, 10.31 piglets), live-born piglets (LBP, 10.31 piglets), number of functional teats (10.94), and shorter average farrowing interval (6 mo). The analysis of sows’ performances based on their origin revealed that sows from agro-ecological zone 8 have the highest number of LBP and the lowest age at first mating and first farrowing. The analysis of sows’ performances based on the hair color showed that those with black hair have the largest LS and the highest number of LBP. This study showed that Benin’s native sows have good reproductive ability with enough variation to develop a sustainable pig industry for a better contribution to food security and wealth creation.

## INTRODUCTION

Demand for animal protein in sub-Saharan Africa has increased with population growth, urbanization, changes in eating habits, and rising living standards (Djimènou, 2019). In Benin, the average annual animal protein consumption was estimated at 12 kg per capita, what is below FAO’s recommendation (20 kg per capita) (FAO, 2015). This gap remains persistent despite substantial meat imports which exceed the annual local meat production, estimated at 187.627 tonnes in 2015 (Dognon et al., 2018).

Therefore, promoting the rearing of animals with a short reproductive cycle, such as pigs (*Sus scrofa*) may help reduce the gap between supply and demand for animal protein in Benin. Native pigs are more prolific than sheep and cattle and have a better ability to survive in harsh conditions (Djimènou, 2019).

Pig meat is one of the most widely consumed meats in Southern Benin because of its organoleptic qualities, especially its flavor (Djimènou et al., 2021). The same authors reported that native pigs and their by-products, apart from their uses as food, play a very important role in culture, religion, traditional medicine, and occultism in Benin. Moreover, the adaptability of native pigs is proven on several levels. Indeed, in terms of health, the native pigs are endowed with a high resistance or tolerance to various diseases contrary to exotic breeds that are highly susceptible to them (Randriamahefa, 2002). It develops a high tolerance to trypanosomiasis, ascariasis, and can survive several epidemics (Adjei et al., 2015; Osei-Amponsah et al., 2017; Ayizanga et al., 2018; Aryee et al., 2019). Thus, the development of pig production is necessary and requires a sound knowledge of the reproductive parameters of the sow.

**Figure 1.**
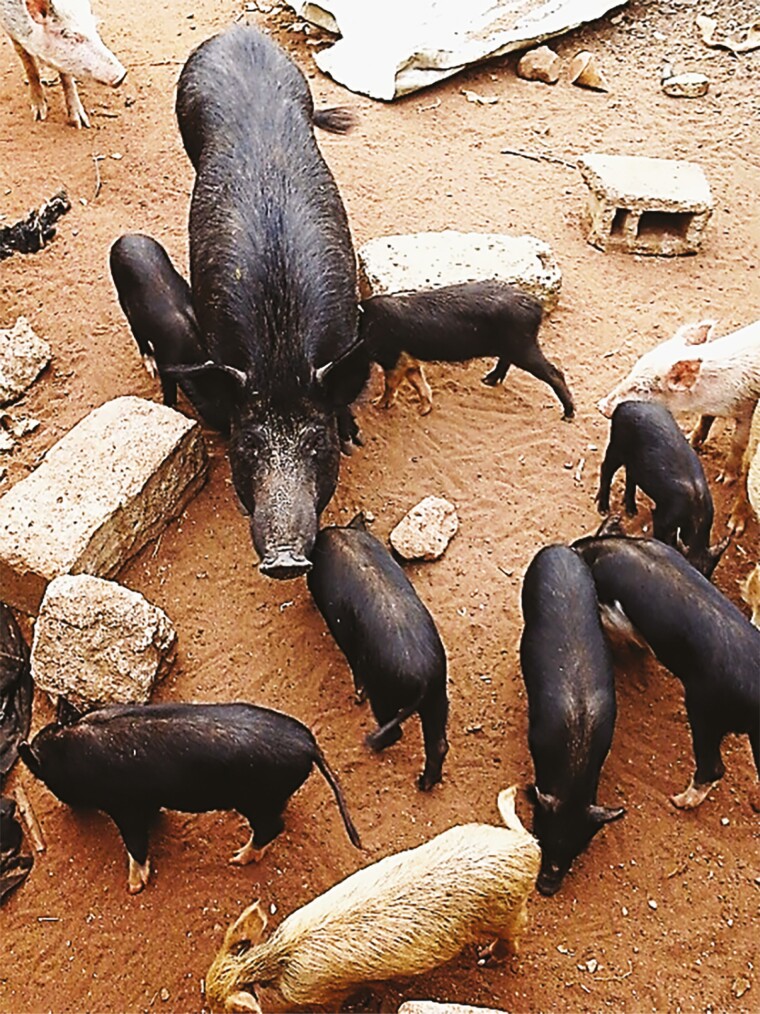
A native sow and its litter in Southern Benin.

Currently, few studies have been carried out on native sows (Figure 1) in semi-confinement breeding system in Benin (Koutinhouin et al., 2009; Youssao et al., 2009a, 2009b). Data on the reproductive performances of native sows reared under extensive farming conditions in Benin remains scanty. However, a good knowledge of the reproductive ability of animal resources is essential for their genetic improvement and sustainable use (FAO, 2013). Hence, this study aims at assessing the best reproductive traits of the native sow breed as a prerequisite for the implementation of the native pig improvement program in Benin.

## METHODOLOGY

The data were collected from field visit and from farmer record after farmer’s agreement was obtained. The data collection did not involve any purpose animal killing or animal movement. Therefore, no ethical approval was necessary.

### Sampling and Data Collection

**Figure 2.**
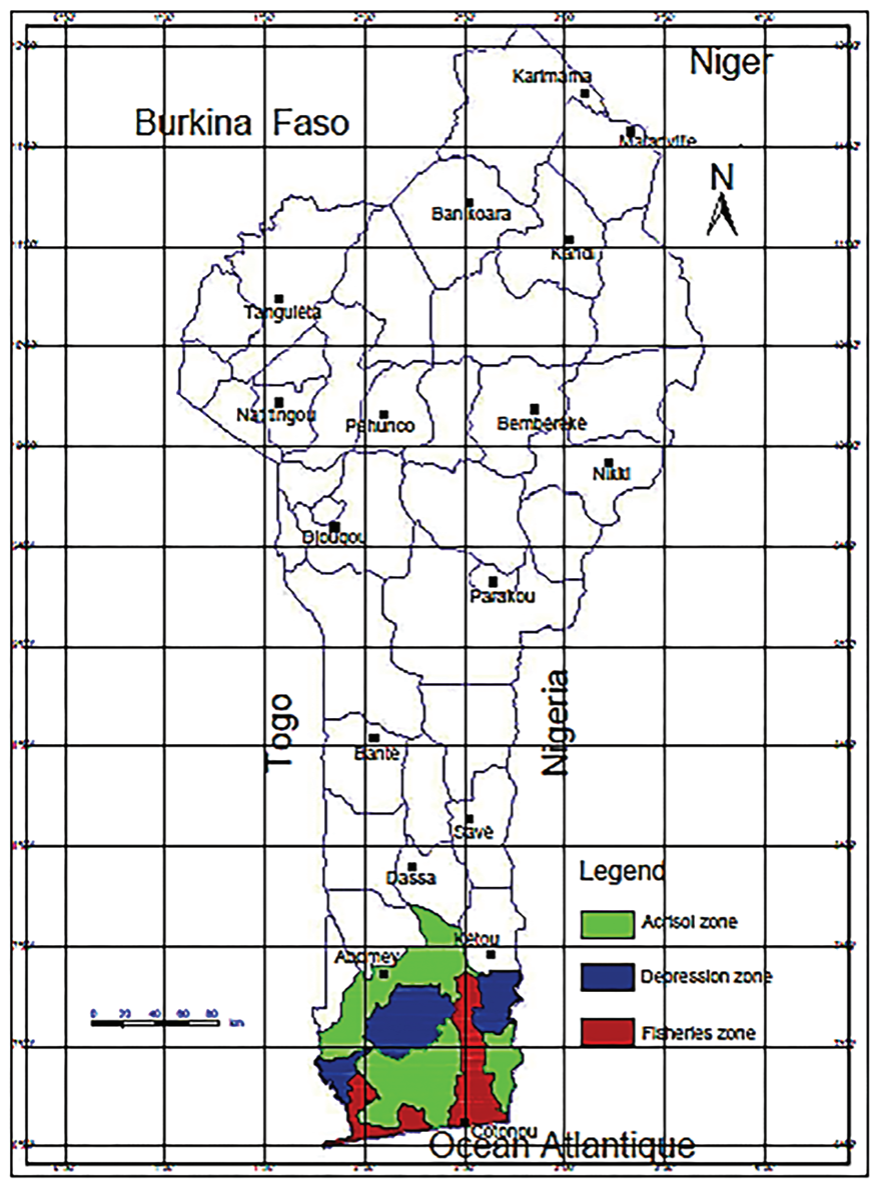
Study areas.

Pig farmers were selected from three of the eight agro-ecological zones in Benin, based on the extent of pig farming in the respective areas (Djimènou et al., 2018). These include (i) zone 6 (Acrisol zone), (ii) zone 7 (Depression zone), and (iii) zone 8 (Fisheries zone) (Figure 2). Data were collected on 284 multiparous native sows from farmers who kept a breeding monitoring sheet. The data collected included sow hair color, litter size (LS), number of live-born piglets (LBP), number of functional teats (TN), age at first mating (AM), age at first farrowing (AF), farrowing interval (FI), number of dead-born piglets (DB), and number of piglets dead before weaning (DW).

### Statistical Analysis

Selected sows were clustered based on their reproductive performances using hierarchical clustering on principal components (HCPC) analysis and the individual reproductive parameters of the sows using *FactoMineR* (Le et al., 2008). The obtained clusters were described by one-factor (cluster) Poisson models and Student–Newman–Keuls (SNK) mean structuring tests (Crawley, 2013) using *agricolae* (de Mendiburu, 2017). Two *stepwise* canonical discriminant analyses were performed to cluster selected sows according to their area of origin, hair color, and reproductive parameters by *klaR* (Weihs et al., 2005). Two further canonical discriminant analyses were then performed to evaluate the discrimination of (i) sow origin (agro-ecological zone) and (ii) sow coat colors based on preselected reproductive parameters by *candisc* (Friendly and Fox, 2017). All these analyses were performed using R software version 3.4.1 (R Core Team, 2017).

## RESULTS

### Discrimination of Sows According to Reproductive Performance

**Figure 3.**
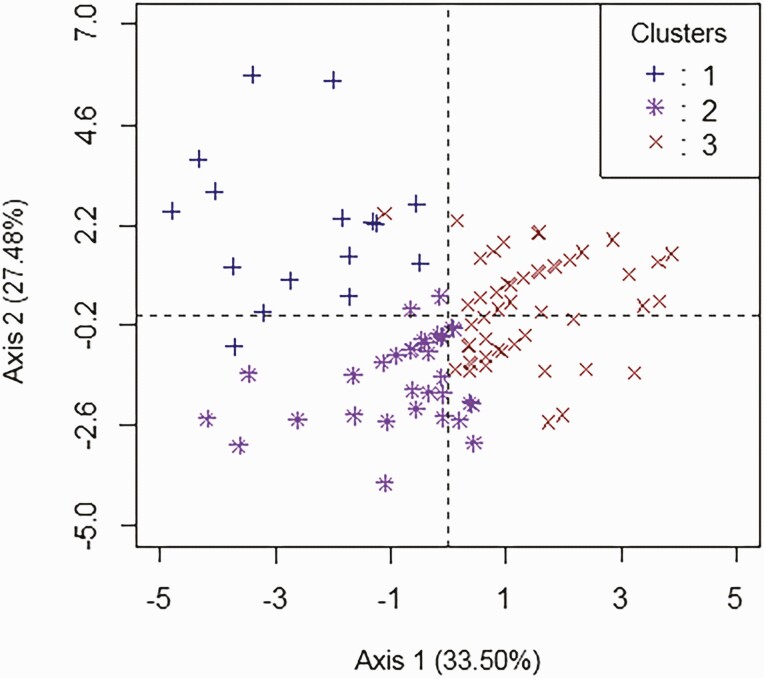
Distribution of the three sow clusters into the 1 and 2 axes HCPC system.

The results of the HCPC showed that the studied population can be distributed into 3 groups including (cluster 1: 30 sows [10.56%]; cluster 2: 107 sows [37.68%], and cluster 3: 147 sows [51.76%], respectively) (Figure 3).

As shown in Table 1, reproductive performance varied significantly (*P* ≤ 0.05) between clusters (*P* ≤ 0.05) with sows in cluster 3 having the best values for LS, LBP, and TN while sows in cluster 2 have the best value for AM, AF, FI, and DB. Sows in cluster 1 have the worse value for all parameters except for DW.

**Table 1.** Characteristics of sow clusters

Cluster	Reproductive parameters							
	LS, piglets	LBP	TN	AM, mo	AF, mo	FI, mo	DB, piglets	DW, piglets
1	7.67^b^ (2.06)	7.33^b^ (1.69)	10.87^a^ (1.46)	9.23^a^ (2.13)	13.23^a^ (2.13)	7.03^a^ (0.85)	0.33^a^ (1.27)	0.50^b^ (0.78)
2	7.53^b^ (1.51)	7.50^b^ (1.55)	9.70^b^ (0.79)	5.76^b^ (0.70)	9.72^b^ (0.79)	5.99^b^ (0.22)	0.04^b^ (0.27)	0.35^b^ (0.70)
3	10.37^a^ (1.22)	10.31^a^ (1.25)	10.94^a^ (1.04)	5.79^b^ (0.75)	9.76^b^ (0.86)	6.00^b^ (0.17)	0.05^b^ (0.33)	1.03^a^ (0.92)
Overall	9.01 (2.01)	8.94 (2.01)	10.46 (1.17)	6.14 (1.44)	10.11 (1.50)	6.11 (0.46)	0.08 (0.51)	0.72 (0.89)
*P*	0.000	0.000	0.008	0.000	0.000	0.104	0.000	0.000

Coefficient of variation values are in parentheses.

Within columns means followed by different superscripts vary significantly at the 5% threshold.

The distribution of sows according to their origin (Table 2) revealed that cluster 1 was dominated by sows from agro-ecological zones 6 and 7, whereas cluster 3 is dominated by sows from agro-ecological zones 8 and 6 (Table 2). Though the majority of sows in cluster 2 are from zone 6, there is also a significant number of sows from zone 8 and zone 7 that fall into this group. Regarding hair color, black was dominant in all clusters (Table 3).

**Table 2.** Distribution of sows according to their origin

Agro-ecological zone	Cluster		
	1	2	3
Zone 6	14 (46.67%)	42 (39.25%)	54 (36.73%)
Zone 7	14 (46.67%)	37 (34.58%)	25 (17.01%)
Zone 8	2 (6.66%)	28 (26.17%)	68 (46.26%)
Overall	30 (10.56%)	107 (37.68%)	147 (51.76%)

Relative frequencies are in parentheses.

Zone 6: Acrisol zone, Zone 7: Depression zone, Zone 8: Fisheries zone.

**Table 3.** Hair color distribution within clusters

Sow hair color	Cluster		
	1	2	3
White	5 (16.67%)	18 (16.83%)	33 (22.45%)
Black	8 (26.67%)	24 (22.43%)	51 (34.69%)
Black with white shoulder belt	2 (6.67%)	18 (16.82%)	14 (9.52%)
Black with white belt on the side	7 (23.33%)	16 (14.95%)	15 (10.20%)
Black with white spots	5 (16.66%)	16 (14.95%)	17 (11.57%)
Magpie	3 (10.00%)	15 (14.02%)	17 (11.57%)

Relative frequencies are in parentheses.

### Discrimination of Agro-Ecological Zones According to the Reproductive Performances of Sows

The results of the stepwise canonical analysis on the reproductive parameters of the sows (Table 4) showed that the number of LBP, the AM, and the AF were the reproductive parameters that discriminate agro-ecological zones.

**Table 4.** Discriminant stepwise analysis on the reproductive parameters of sows

Number of steps	Variable	*F*	Pr > *F*	Wilk’s lambda	Pr > Lambda
1	LBP	15.02	0.000	0.903	0.000
2	AM	6.36	0.002	0.864	0.000
3	AF	2.17	0.116	0.851	0.000

Table 5 presents the results of the inferential tests and the mean characteristics of the sows for the discriminating variables according to the agro-ecological zones. It showed a concordance of the results of the inferential tests and the stepwise canonical discriminant analysis, confirming the discriminating power of the agro-ecological zones on LBP, AM, and AF (*P* < 0.05).

**Table 5.** Variation of discriminating reproductive parameters of sows according to agro-ecological zones

Agro-ecological zone	AM, mo	AF, mo	LBP
Zone 6	6.24^a^ (1.63)	10.15^b^ (1.76)	8.99^b^ (1.97)
Zone 7	6.59^a^ (1.61)	10.59^a^ (1.61)	8.00^c^ (2.16)
Zone 8	5.67^b^ (0.79)	9.68^c^ (0.79)	9.60^a^ (1.62)
Overall	6.14 (1.44)	10.11 (1.50)	8.94 (2.00)
*P*	0.000	0.000	0.001

Values in parentheses are SD.

Within columns means followed by different superscripts vary significantly at the 5% threshold.

The average of the reproductive parameters of the sows (Table 5) indicates that their AM and AF in agro-ecological zone 8 were lower (1 mo less on average) than values obtained in agro-ecological zones 6 and 7. On the other hand, LBP was higher in agro-ecological zone 8 than in zones 6 and 7 by one and two units, respectively. It is worth mentioning that sows in agro-ecological zone 7 have the highest AM and AF and the lowest LBP.

**Figure 4.**
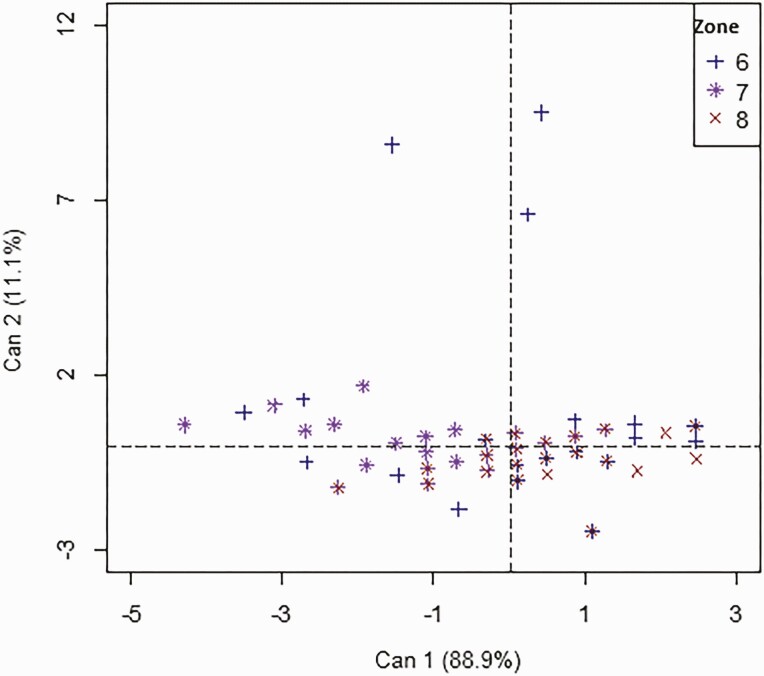
Discrimination of local sows according to reproductive parameters using agro-ecological zones in the axes system.

The canonical discriminant analysis performed on the reproductive parameters of the sows according to the three agro-ecological zones investigated (Table 6) indicated that canonical axis 1 includes all three reproductive parameters. Thus, AM and AF negatively correlated with this axis showed that the sows in zone 7 have high values for these two parameters. In contrast, the sows in zone 8 had high values for the number of LBP (Figure 4).

**Table 6.** Canonical discriminant analysis of the reproductive parameters of sows according to agro-ecological zones

Variables	Canonical axes	
	1	2
Sows’ reproductive parameters		
AM	−0.690	0.298
AF	−0.651	0.058
LBP	0.845	0.311
Agro-ecological zones		
Zone 6	−0.033	0.172
Zone 7	−0.542	−0.123
Zone 8	0.458	−0.098

### Hair Color Discrimination of Sows Based on Their Reproductive Performances

The results of the stepwise discriminant analysis of the reproductive parameters and hair color of sows (Table 7) showed that LBP and LS were the parameters that discriminated the hair color of sows on the basis of Wilk’s lambda statistics and associated probability value.

**Table 7.** Discriminant step-by-step analysis of the reproductive parameters of sows according to hair color

Number of steps	Variables	*F*	*P* > *F*	Wilk’s lambda	*P* > Lambda
1	LBP	0.017	0.952	0.017	0.010
2	LS	0.100	0.921	0.011	0.016

Table 8 presents the results of Poisson regressions and the trends of the discriminant reproductive parameters according to the sow’s hair color. The analysis of the reproductive parameters revealed that globally there are two clusters of sows: the first cluster is composed of black, white, and magpie hair and the second cluster consists of black and white spots, black with a white belt on the flank and black with a white belt on the shoulder hair. Irrespective of the reproductive parameter considered, the first sow cluster shows the best performance.

**Table 8.** Reproductive characteristics of sows according to dress color

Dress color	LBP, piglets	LS, piglets
Black	9.34^a^ (0.27)	9.41^a^ (0.21)
White	9.29^a^ (0.34)	9.38^a^ (0.27)
Magpie	9.14^a^ (0.28)	9.14^a^ (0.33)
Black with white spots	8.45^b^ (0.21)	8.71^b^ (0.38)
Black with white belt on the side	8.42^b^ (0.37)	8.45^bc^ (0.28)
Black with white shoulder belt	8.29^b^ (0.33)	8.29^c^ (0.34)
Overall	8.94 (2.01)	9.01 (2.01)
*P*	0.015	0.016

Values in parentheses are SD.

Within columns means followed by different superscripts vary significantly at the 5% threshold.

## DISCUSSION

The present study, on the basis of a selected number of reproductive parameters (LS, LBP, and AM), reveals the existence of three clusters among the native sow breed in Southern Benin. The LS varies significantly between clusters and can reach up to 10 piglets on average. This value is above those reported in most previous studies carried out in Benin and other sub-Saharan African countries. This variation could be due to the difference in breeding practices, feeding, environment, and genetic types (Ranjit et al., 2015). In Benin, the reported average LS of the native sow in the traditional breeding system was 5.74 piglets (Atodjinou and Dotcho, 2006). In the improved breeding system, recorded values fell between 6.31 and 8.8 piglets (Koutinhouin et al. 2009; Youssao et al., 2009a, 2009b; Agbokounou et al., 2016; Dotché et al., 2020). Likewise, d’Orgeval (1997) reported closer value in Nigeria (6.7) and Cameroon (7.8), respectively. The average LS obtained in this study (9.1) is close to the value recorded from exotic breeds reared in the improved breeding system in Southern Benin (9.7) (Youssao et al., 2009a) but also in their original environment (9–10.5) (Nowak et al., 2020). This implies that the reproductive performances of the native sows can be better if rearing conditions are improved (Djimènou, 2019). The number of average LBP in the present study was higher than 5.3 recorded in native sows of Bangladesh (Motaleb et al., 2014). The Prolificacy of native sows in Southern Benin was a criterion for the national breeding program aiming at increasing the numerical productivity of native sows in Benin. It was noticed that sows with the highest number of teats have the highest LS. Thus, the TN could be used as an indicator to detect potential prolific sows.

The AM (6 mo) was similar to that recorded in improved pig breeding system (Youssao et al., 2009a, 2009b) and lower than that recorded in the traditional breeding system (9 mo) (Dotché et al., 2018) in Ouémé and Plateau departments in Benin but also than that recorded in Bangladesh native sows (7.5 mo) (Motaleb et al., 2014). Thus, the present study showed that the genetic potential of the native sows in terms of sexual maturity is not fully utilized yet in Benin.

The AF (10.11 mo on average) and the FI (6 mo) obtained were similar to recorded values of 10.43 mo and 6.09 mo, respectively, in Bangladesh native sows (Ritchil et al., 2014). Moreover, the AF observed was less than 11.88 mo reported in native sows in Ouémé and Plateau departments in Benin (Dotché et al., 2020) and 12.5 mo reported in Indian native sows (Singh et al., 2020). In contrast, AF in the present study was higher than that of 9.1 mo in Nigerian pigs (Abah et al., 2019). Based on their FI, native sows can farrow twice a year, which demonstrates the profitability of pig farming compared to sheep, goat, and cattle farming. The mortality rate from birth to weaning (8% in average) was 2.5 times lower than the rate reported in large-white sows bred in intensive systems (Youssao et al., 2009a). Overall, the observed differences can be explained by various reasons. Firstly, as reported previously, data were collected from semi-intensive pig farms. Thus, these breeders are better organized in terms of rearing practices, which could guarantee a better performance. Pigs, especially native pigs, are omnivores whose digestion physiology is very well adapted to the valorization of kitchen waste that is reported to have poor nutritional value (Djimènou et al., 2017). The use of these kitchen wastes can make feeding cheaper while boosting the animal performances. But it is important to guarantee the food safety to avoid food-borne disease transmission. Secondly, the sows included in our study were all multiparous. Indeed, LS increases as farrowing number increases (Youssao et al., 2009a).

The heterogeneous distribution of sows within agro-ecological zones for all traits is in agreement with the previous results of the phenotypic characterization of native pigs (Djimènou et al., 2018). However, the discriminant analysis identified three sow reproductive parameters such as LS, AM, and AF which differ significantly across zones. Thus, native sows in Benin have a good reproductive ability that can be improved for their conservation and sustainable use for protein self-sufficiency and wealth creation in Benin.

## CONCLUSION

This study reported on the reproductive performances of native sows reared in extensive farming systems in Southern Benin. The existence of great variations in some reproductive parameters such as LS, at-birth viability of piglets, AM, is a good asset for selecting individuals with higher performances in order to improve productivity at the herd level. Additionally, to ensure a sustainable management of native sows, it is important to use molecular genetic characterization tools such as marker-assisted selection and single-nucleotide polymorphism genotyping to improve the accuracy of selection and hasten their genetic improvement.
